# Phytotoxins Produced by Two *Biscogniauxia rosacearum* Strains, Causal Agents of Grapevine Trunk Diseases, and Charcoal Canker of Oak Trees in Iran

**DOI:** 10.3390/toxins13110812

**Published:** 2021-11-18

**Authors:** Marco Masi, Samaneh Bashiri, Alessio Cimmino, Zeinab Bahmani, Jafar Abdollahzadeh, Antonio Evidente

**Affiliations:** 1 Departiment of Chemical Sciences, University of Naples Federico II, Complesso Universitario Monte S. Angelo, Via Cintia 4, 80126 Napoli, Italy; marco.masi@unina.it (M.M.); alessio.cimmino@unina.it (A.C.); 2 Department of Plant Protection, Agriculture Faculty, University of Kurdistan, P.O. Box 416, Sanandaj 66177-15175, Iran; sbashiri2013@gmail.com (S.B.); z_bahmani65@yahoo.com (Z.B.)

**Keywords:** *Biscogniauxia rosacearum*, grapevine, oak trees, phytotoxins, isocoumarines, SAR studies

## Abstract

*Biscogniauxia rosacearum,* recognized for the first time as a pathogen involved in grapevine trunk diseases in Paveh (west of Iran) vineyards, produced *meso*-2,3-butanediol (**1**) as the only phytotoxin. Nectriapyrone (**2**), (3*R*)-5-methylmellein (**3**), (3*R*)-5-methyl-6-methoxymellein (**4**), and tyrosol (**5**) were instead produced as phytotoxins from a strain of the same fungus isolated from oak trees in Zagros forests of Gilan-e Gharb, Kermanshah Province. They were identified comparing their ^1^H and ^13^C NMR, ESIMS, and specific optical rotation data with those already reported in the literature. The phytotoxicity of metabolites (**1–5**) was estimated by leaf puncture assay on *Quercus ilex* L. and *Hedera helix* L., and by leaf absorption assay on grapevine (*Vitis vinifera* L.) at a concentration of 5 × 10^−3^ and 10^−3^ M. Tested on grapevine, *meso*-2,3-butanediol (**1**) and (3*R*)-5-methyl-6-methoxymellein (**4**) resulted to be the most phytotoxic compounds. On *Q. ilex*, nectriapyrone (**2**) and tyrosol (**5**) showed severe necrosis at the highest concentration while none of the compounds (**1**–**5**) was active on *H. helix*. Furthermore, the phytotoxicity of compounds **3** and **4** was also compared with that of some related natural melleins to perform a structure-activity relationship (SAR) study. The results of this study were also discussed.

## 1. Introduction

Among the biotic stress agents that affect grapevine (*Vitis vinifera* L.), causing severe diseases with significant losses of wine production yield, there are the fungi responsible for grapevine trunk diseases (GTDs: of esca, eutypiosis, and Botryosphaeria dieback). Different fungi are involved in GTDs such as *Phaeoacremonium, Phaeomoniella, Neofusicoccum, Diplodia, Lasiodiplodia, Eutypa, Dothiorella, Spencermartinsia,* and *Diaporthe* spp. etc., which produce different phytotoxins such as polyketides, isocoumarins, cyclohexene epoxide, chromanones, phenols, dihydrofuranones, quinones, jasmonic acid esters, aromatic compounds, etc. [1,2,3,4,5,6,7,8,9,10].

Recently, massarilactones D and H were isolated for first time as phytotoxins produced by *Kalmusia variispora*, responsible for GTDs in Iran [11]. The symptoms of the same disease in Iran were also induced by *Didymella glomerata* and *Truncatella angustata,* which synthesized the phytotoxic 5-dihydroxymethylfuran and (+)-6-hydroxyramulosin respectively [12]. *T. angustata* also produced, probably as an antagonist, phenazine-1-carboxylic acid (PCA), which showed antifungal activity against four different fungi responsible for GTDs: *Phaeoacremonium minimum, Phaeoacremonium italicum,* and *Fomitiporia mediterranea*, involved in grapevine esca disease, and *Neofusicoccum parvum*, responsible for Botryosphaeria dieback [13].

*Quercus* species (oak cork, ash, mogany etc.) in different world regions have been heavily affected by fungal disease, causing severe economic losses to nurseries and to the wood producers, such as those caused by oak cork, ash, mogany etc. as well as forests and ornamental gardens. The fungal agents belonging to different genera such as *Diplodia, Sphaeropsis, Seiridium, Neofusicoccum, Discula, Cryphonectria, Ophiostoma, Ceratocystis*, *Biscogniauxia* etc., produce several phytotoxins as furanones, terpenes, cyclohexene oxide, isocumarins, pyrones, aromatic compound, polyketides, steroides derivatives, polysaccharides etc. [14].

Recently, *Hymenoscyphus fraxineus* responsible for ash (*Fraxinus excelsior* L.) dieback in Europe was recognized, and the phytotoxic hyfraxinic acid was isolated together with the already known 1-deoxyviridiol, nodulisporiviridin M, viridiol and demethoxyviridiol; the last two metabolites also showed phytotoxic activity [15]. The phytotoxic olicleistanone; (3*R*)-mellein; sphaeropsidins A, C, and G; and diplopimarane were synthesized by fom *Diplodia olivarum*, which is the causal agent of branch canker and dieback of olive drupes in southern Italy, carob tree, and wild olive [16]. Similarly, the phytoxic rabenchromenone and rabenzophenone were isolated from *Fimetariella rabenhorstii*, causing oak decline in Iran [17], while the 5-hydroxymethylfuraldehyde, 2,5-dihydroxymethylfuran, and tyrosol were isolated from *Stilbocrea macrostoma*, inducing necrosis and declining symptoms on *Quercus brantii* trees in the same country [18].

*Cryphonectria parasitica*, the causal agent of chestnut blight [19], and *Pestalotiopsis guepinii* [20,21] and *Diaporthella cryptica* [22], the causal agents of chestnut blight and hazelnut cankers, produced phytotoxic anthraquinones and polysaccharide and phytotoxic pyrones and methyl ester of (*S*)-phenyllactic acid, respectively.

During an extensive survey to characterize fungi associated with grapevine trunk diseases in Kermanshah Province (west of Iran) vineyards, 286 isolates were collected from the infected tissues. Of these, 16 isolates, on the basis of morphology, cultural characteristics, and molecular data (ITS sequence data), were characterized as *Biscogniauxia rosacearum*. Thus, this fungus was reported for the first time as a pathogen associated with grapevine trunk diseases across the world [23].

Similarly, investigation allowed to obtain 500 fungal isolates from twigs and trunks of oak trees showing decline symptoms in Zagros forests, west of Iran. Of these, 57 isolates, based on morphology, cultural characteristics, and sequence data (ITS and β-tubulin) of representative isolates, were characterized as *Biscogniauxia rosacearum* and *Obolarina persica*. Following Koch’s postulates, pathogenicity of both species was confirmed on oak seedlings in greenhouse conditions (Bashiri et al. 2021 private communication).

Thus, the two strains of *B. rosacearum* isolated from infected grapevine trunk, and oak trees were grown in vitro to evaluate their ability to produce phytotoxic metabolites involved in the plant–pathogen interaction.

This work describes the isolation and chemical and biological characterization of the phytotoxins produced by the two strains of *B. rosacearum* and the results of a structure–activity relationships (SAR) study performed with natural melleins, some of which were produced from the strain pathogen on oak tree.

## 2. Results and Discussion

**Figure 1:**
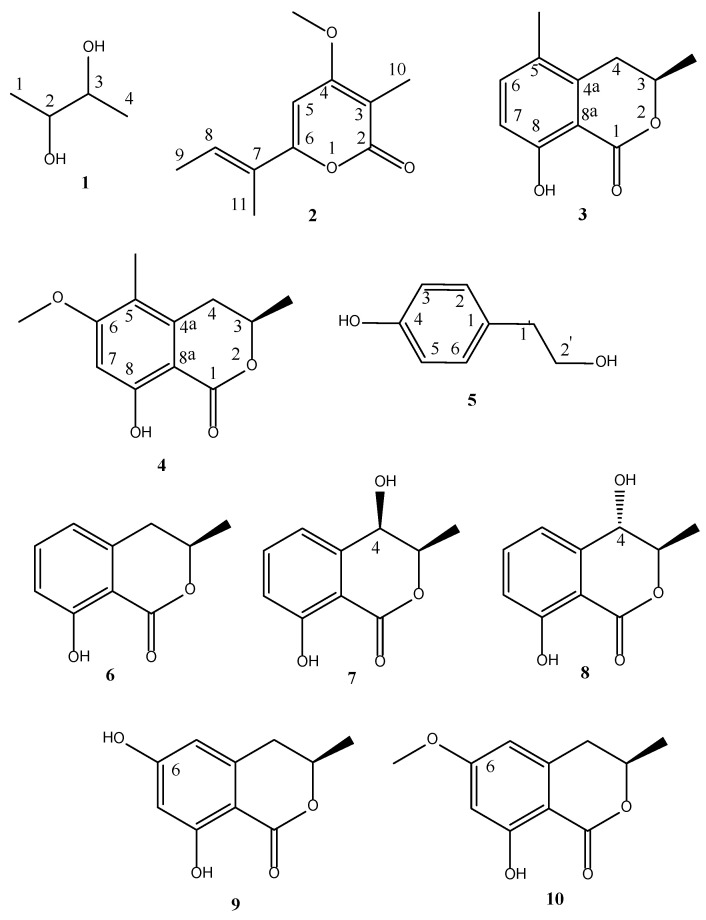
Structures of: *meso*-2,3-butanediol (**1**) isolated from *B. rosacearum* IRAN 4194C; nectriapyrone (**2**), (3*R*)-5-methylmellein (**3**), (3*R*)-5-methyl-6-methoxymellein (**4**), and tyrosol (**5**) isolated from *B. rosacearum* IRAN 4287C; and (3*R*)-mellein (**6**), (3*R*,4*R*)-4-hydroxymellein (**7**), (3*R*,4*S*)-4-hydroxymellein (**8**), (3*R*)-6-hydroxymellein (**9**), and (3*R*)-4-methoxymellein (**10**).

*B. rosacearum* strains IRAN 4194C and IRAN 4287C were grown in vitro, and their culture filtrates were extracted with EtOAc. The preliminary investigation of their organic extracts revealed a substantial difference in the toxins production. Thus, these organic extracts were fractionated, (see Material and Methods Section) to give five homogeneous metabolites. The grapevine strain of *B. rosacearum* IRAN 4194C produced only one main metabolite, while the other four compounds were isolated from the oak strain *B. rosacearum* IRAN 4287C. They were identified as *meso*-2,3-butanediol, nectriapyrone, (3*R*)-5-methylmellein, (3*R*)-5-methy-6-methoxymellin, and tyrosol (**1**–**5**, Figure 1) by comparison of their ^1^H and **^13^**C NMR and ESI MS spectra, and were needed the specific optical rotations with the data already reported in literature (see below).

In particular, the ^1^H NMR of **1**, a symmetric compound**,** showed the quartet (*J* = 6.5 Hz) at δ 3.80, typical of the protons (H-2 and H-3) bonded to a secondary hydroxylated carbon, which coupled with the protons of the adjacent methyl group (H_3_-C1 and H_3_-C4) resonating as a doublet (*J* = 6.5 Hz) at δ 1.14 [24]. The ^13^C NMR spectrum showed the signals of the oxygenated carbons (C-2 and C3) at the typical values of δ 70.8 and those of the two methyl carbons (C-1 and C-4) at δ 16.8 [25]. The ESI MS spectrum showed the significant ions generated from the protonated molecular adduct ion by loss of water [M +H - H_2_O]^+^ at *m*/*z* 73. Furthermore, compound **1** did not show optical activity, thus it was identified as the *meso*-butanediol (**1**), whose spectroscopic data were in agreement with those reported by Gallwey et al., 1990 [26].

2,3-Butanediol is a well-known fungal and bacterial metabolite [27,28,29,30] and is used for important biotechnological applications to produce liquid fuel and chemical raw materials. In fact, 2,3-butanediol and its derivatives have broad industrial application prospectives [31,32]. Thus, its production in high yield by fungal fermentation is very suitable to develop such biotechnological applications. 2,3-Butanediol have three stereoisomers: dextro- [L−(+)−] and levo-[D−(−)−] forms, both optically active, and the optically inactive *meso*-form as **1**. The steochemistry of the produced 2,3-butanediol depends on the microorganism producer; however, the *meso*-form is the most common stereoisomer [28,29].

The ^1^H NMR spectrum of nectriapyrone (**2**) showed the quartet (*J* = 7.2 Hz) of an olefinic proton (H-8) at δ 6.69, which coupled with the protons (H_3_-9) of the adjacent vinyl methyl, resonating as a doublet (*J* = 7.2 Hz) at δ 1.84; a singlet of another olefinic proton (H-5) at δ 6.09; and three singlets at δ 3.90 (OMe), 1.94 (H_3_-C7), and 1.89 (H_3_-C3) due to a methoxy and two methyl vinyl groups [24]. The ^13^C NMR spectrum showed the signals of the ester carbonyl (C-2) and the two protonated olefinic carbons (C-8 and C-5) at δ 165.2, 129.9, and 91.6, respectively. The signals of the methoxy and the three methyl groups appeared at δ 56.2 (OMe), 14.4 and 12.3 (C-9/C-11 or C11/C9), and 8.8 (C-10), respectively. The singlets of four tertiary sp^2^ carbons, two of which are oxygenated, resonated at δ 166.1 (C-4), 160. 3 (C-6), 127.1 (C-7), and 102.1 (C-3) [25]. Its ESI MS spectrum exhibited the dimer sodiated [2M + Na]^+^, the sodiated [M + Na]^+^, and the protonated [M + H]^+^ adduct ions at *m*/*z* 411, 217, and 195, respectively. These data were in agreement with those previously reported [9,33,34].

Nectriapyrone (**2**) was previously isolated as phytotoxin produced by phytopatogenic fungi as *Diaporthe angelicae* (anamorph *Phomopsis foeniculi*), which is the causal agent of fennel diseases (*Foeniculum vulgare*) in Bulgaria [34]. Compound **2** was also isolated from *Pestalotiopsis guepinii*, which induced hazelnut twig blight [20,21] and recently by *Diaporthe eres* which was involved in the GTDs symptoms [9].

(3*R*)-5-Methylmellin was characterized by ^1^H and ^13^C NMR and ESI MS spectra, but also by measuring its specific optical rotation, which is in agreement with the value previously reported by Okuno et al., 1986 [35]. In particular, its ^1^H NMR spectrum showed a singlet at δ 10.98 due to a hydroxyl group at C-8 hydrogen bonded with the C-1 carbonyl group and two doublets (*J* = 8.4 Hz) at δ 7.28 and 6.82, which are typical signals of two *ortho-*coupled aromatic protons (H-6 and H-7, respectively) of a tetrasubstituted benzene ring with H-7 hupfield shifted for the electronic effect of the *ortho*-located HO-C8. In addition, a multiplet of a proton (H-3) of a secondary oxygenated carbon appeared at δ 4.69, which coupled with the protons of the adjacent methylene group (H_2_C-4), resonating as two double doublets (*J* = 16.5 and 2.0 Hz and *J* =16.5 and 11.9 Hz) at δ 2.95 and 2.72. H-3 also coupled with the protons of the geminal methyl group (Me-C3), appearing as a doublet (*J* = 7.2 Hz) at δ 1.84 [24]. The ^13^C NMR spectrum showed the singlet of the ester carbonyl group (O = C-1) at δ 170.1 together with the signals of the protonated secondary carbons, two of which are aromatic (C-6 and C-7) and the other one aliphatic (C-3) at δ 137.4, 115.2, and 74.8. C-7) was up-field shifted for the electronic effect of the HOC-8,. The carbons of the methylene group (C-4), those of the vinylic methyl group (Me-C5) and of the aliphatic one (MeC-3) were observed at δ 31,5, 16.2 and 20,6, respectively. The aromatic tertiary sp^2^ carbons, one which was oxygenated, resonated at δ 160.0, 137.6 137.3, and 108.3 for C-8, C-5, C-4a, and C-8a, with the last up-field shifted as reported for C-7 [25]. Its ESI MS spectrum showed the protonated adduct ion [M + H]^+^ at *m*/*z* 193. These data were in agreement with those reported by Okuno et al., 1986 [35].

(3*R*)-5-methyl-6-methoxymellein (**4**) showed a specific optical rotation in agreement to the value reported by de Alvarenga et al., 1978 [36]. It was identified by ^1^H and ^13^C NMR and ESI MS data. In particular, its ^1^H NMR spectrum showed the singlet of the HO-C 8 at δ 11.36 being hydrogen bonded, as in **3**, with the carbonyl group at C- 1 and another singlet typical of a proton (H-7) of a pentasubstituted benzene ring at δ 6.38. The latter signal was upfield shifted for the electronic effect of the *ortho*-hydroxyl group at C-8. The same spectrum showed the other two singlets at δ 3.85 and 2.03, typical of a methoxy group and a vinyl methyl (MeC-5) together with the multiplet of the proton (H-3) of an oxygenated secondary carbon resonating as a multiplet at δ 4.61. H-3 coupled with the protons of the adjacent methylene group (H_2_C-4) and those of germinal methyl group (Me-C3), which were observed as two double doublets (*J* = 16.7 and 2.0 Hz, and *J* = 16.7 and 11.9 Hz) and as a doublet (*J* = 6.3 Hz) at δ 2.97, 2.68 and 1.56, respectively [24]. The ^13^C NMR spectrum showed the singlet of the ester carbonyl group (C-1) and the signals of secondary carbons (C-7 and C-3) and of the methylene group (H_2_C-4) at δ 115.1, 74.8, and 31.9. The signals of the methoxy group (OMe), of the vinyl methyl (Me-C5), and of the aliphatic one (Me-C3) were observed at δ 55.7, 10.6, and 20.9, respectively. The five aromatic tertiary sp^2^ carbons, two of which were oxygenated, appeared at δ 164.6, 162.9, 137.6, 137.4, and 114.7 for C-6, C-8, C-5, C-4a, and C-8a. This latter signal appeared upfield shifted for the electronic effect of the *ortho-*located hydroxyl group at C-8, which similarly affects the chemical shift of C-7 [25]. The ESIMS spectrum showed the dimer sodiated [2M + Na]^+^ and the protonated [M + H]^+^ adduct ions at *m*/*z* 467 and 223. These data are in agreement to those previously reported by de Alvarenga et al., 1978 [36].

The *ortho*-location of the methoxy group and the vinyl methyl at C-6 and C-5, respectively, was determined by the correlations observed in the NOESY spectrum [37] between the methoy group with H-7 and that of Me-C5 with H_2_-C-4. The correlation between the methoxy group with MeC-5 was probably not observed as the first is oriented toward H-7 and its rotation is almost hindered.

(3*R*)-5-methyl and (3*R*)-5-methyl-6-methoxy-mellein (**3** and **4**) both belong to the group of 4-dihydroisocoumarins and are very well known as naturally occurring compounds. They are important metabolites for the producer organisms and are involved in many biological activities including phytotoxicity [38].

Tyrosol (**5**) was identified by comparing its ^1^H and ^13^C NMR and ESIMS data with those previously reported Reveglia et al., 2021 [9]. In particular, its ^1^H NMR spectrum showed a couple of doublets (*J* = 8.0 Hz) of the two aromatic *ortho*-coupled protons of a *p*-disubstituted benzene ring at δ 7.20 (H-2 and H-6) and 6.80 (H-3 and H-5) with the last signal upfield shifted for the electronic effect of the hydroxyl group *ortho*-located at C-4. The same spectrum showed the two triplets (*J* = 6.4 Hz) of the two methyelene groups of 2-hydroxy ethyl side chain observed at δ 3.80 (H_2_C-2’) and 2.80 (H_2_C-1’) [24]. The ^13^C NMR spectrum showed the overlapped signals of the protonated aromatic carbons (C2/C6) and (C3/C5) at δ 113.0 and 118.5, with the latter upfield shifted for the electronic effect already described by the hydroxyl group at C-4, and the two methylene carbons of the side chain at δ 65.4 and 39.6 for C-2’and C-1’, respectively. The two tertiary sp^2^ aromatic carbons, one of which is oxygenated, resonated at δ 156.5 and 133.6, for C-4 and C-1, respectively [25]. It ESIMS spectrum exhibited the dimer sodiated [2M + Na]^+^ and the sodiated [M + Na]^+^ adduct ions at *m*/*z* 299 and 139, respectively. These data were in agreement to those previously reported [9].

Tyrosol (**5**) is a phytotoxin produced by both plants [39] and fungi as the grapevine pathogenic fungi *Lasiodiplodia euphorbicola*, *Lasiodiplodia hormozganensis* [40], *Neofusicoccum australe*, and [41] and *N. parvum* [42]. It was also produced by *Diplodia seriata* (syn. *Botryosphaeria obtusa*), a pathogen of apple fruit and frogeye leaf [43], and it is toxic to tomato and is a quorum sensing molecule in *Candida albicans* [44].

**Figure 2:**
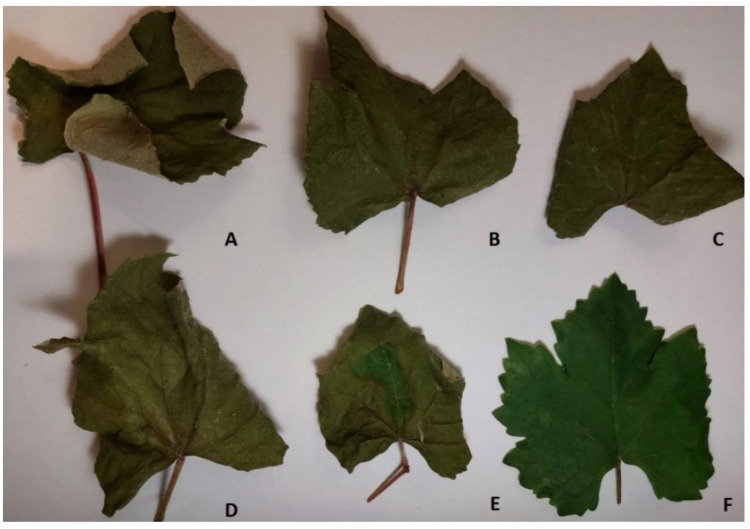
Phytotoxic activity induced by *meso*-2,3-butanediol (**1**) (**A**), nectriapyrone (**2**) (**B**), (3*R*)-5-methylmellein (**3**) (**C**), (3*R*)-5-methyl-6-methoxymellein (**4**) (**D**), and tyrosol (**5**) (**E**) at 10^−3^ M by leaf absorption assay on grapevine. Negative control is 4% MeOH in MilliQ H_2_O (**F**).

**Figure 3:**
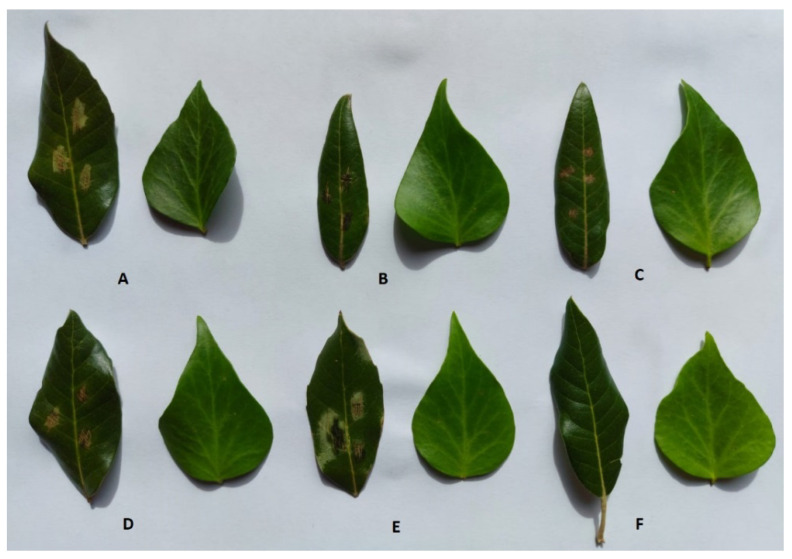
Phytotoxic activity observed by testing by *meso*-2,3-butanediol (**1**) (**A**), nectriapyrone (**2**) (**B**), (3*R*)-5-methylmellein (**3**) (**C**), (3*R*)-5-methyl-6-methoxymellein (**4**) (**D**), and tyrosol (**5**) (**E**) at 10^−3^ M using leaf puncture assay on *Q. ilex* L. (left leaves) and *H. helix* L. (right leaves). Negative control is 4% MeOH in MilliQ H_2_O (**F**).

The phytotoxic activity of compounds (**1**–**5**) were estimated by leaf puncture assay on *Quercus* ilex L. and *Hedera helix* L., and by leaf absorption assay on grapevine (*Vitis vinifera* L.) at a concentration of 5 × 10^−3^ M and 10^−3^ M. The results of these assays are reported in Table 1. In the leaf absorption assay, *meso*-2,3-butanediol (**1**) and (3*R*)-5-methyl-6-methoxymellein (**4**) resulted to be the most phytotoxic compounds (Figure 2). In the leaf puncture assays, nectriapyrone (**2**) and tyrosol (**5**) induced severe necrosis at the highest concentration while none of the compounds (**1**–**5**) were active on *H. helix* L (Figure 3).

Furthermore, the activity of compounds **3** and **4** was also compared with that of some related melleins, namely (3*R*)-mellein (**6**) and (3*R*,4*R*)-and (3*R*,4*S*)-4-hydroxy melleins (**7** and **8**), isolated fom *Sardiniella urbana,* [45] and (3*R*)-6-hydroxymellein (**9**), isolated from *Phoma chenopodiicola,* as previously described [46]. Also, the (3*R*)-6-methoxymellein (**10**), which was prepared by methylation starting from **9** by reaction with an ether solution of diazomethane, was used. The results of this structure–activity relationship (SAR) study, reported in Table 1, showed that in the toxicity on grapevine, the hydroxy group at C-4 of pyranone moiety negatively affected the phytotoxicity on *V. vinifera* L. Instead, the C-6 substitution of the aromatic ring either with a phenolic hydroxy or a methoxy group is an important feature to cause phytotoxicity on the same plant. The results obtained on *Quercus ilex* L. (Table 1) suggested that the absence of any substituents on the aromatic ring is essential feature for the toxicity, demonstrating a different mode of action of the melleins on grapevine and oak leaves.

## 3. Conclusions

*meso*-2,3-Butanediol (**1**) is the only phytotoxin synthesized by *Biscogniauxia rosacearum* (IRAN 4194C)*,* which was recognized for the first time as a pathogen involved in GTDs in Paveh, Kermanshah Province (west of Iran) vineyards. Similarly, nectriapyrone (**2**), (3*R*)-5-methylmellein (**3**), (3*R*)-5-methyl-6-methoxymellein (**4**), and tyrosol (**5**) were instead produced as phytotoxins from a strain of the same fungus (IRAN 4287C) isolated from oak trees in Zagros forests of Gilan-e Gharb, Kermanshah Province. Tested on grapevine (*Vitis vinifera* L.), *meso*-2,3-butanediol and (3*R*)-5-methyl-6-methoxymellein resulted to be the most phytotoxic compounds, while nectriapyrone (**2**) and tyrosol (**5**) showed severe necrosis at the highest concentration when assayed on oak (*Quercus ilex* L.) leaves. On ivy (*H. helix* L), none of the compounds (**1**–**5**) were active. The results of SAR study using melleins **3**,**4** and **6**–**10** showed that the hydroxy group at C-4 of pyranone ring negatively affected the phytotoxicity on *V. vinifera* L., while the C-6 substitution of benzene ring either with a phenolic hydroxy group or a methoxy group is determinant for the phytotoxicity. On *Quercus ilex* L., the absence of any substituents on the aromatic ring is an essential feature to impart phytotoxic activity. These results suggested a different mode of action of the melleins on grapevine and oak leaves. Further studies need to be performed on the phytotoxins/plant–pathogen interaction.

## 4. Materials and Methods

### 4.1. General Experimental Procedures

The optical rotations were recorded on a JASCO P-1010 (Tokyo, Japan) digital polarimeter. A spectrometer 400 MHz Bruker was employed to record ^1^H NMR spectra in CDCl_3_ or CD_3_OD, which were used also as internal standards. TOF LC/MS spectrometer Agilent 6230B was used to record ESI mass spectra. Analytical and preparative Thin-Layer Chromatography (TLC) was performed on SiO_2_ plates (Kieselgel 60, F_254_, 0.25 and 0.5 mm respectively) or on reverse phase (Whatman, KC18 F_254_, 0.20 mm) plates (Merck, Darmstadt, Germany). The spots were visualized by exposure to UV light (254 nm) and/or iodine vapors and/or by spraying first with 10% H_2_SO_4_ in MeOH, and then with 5% phosphomolybdic acid in EtOH, followed by heating at 110 °C for 10 min. Column chromatography was carried out using silica gel (Merck, Kieselgel 60, 0.063–0.200 mm). The samples of (3*R*)-mellein (**6**); (3*R*,4*R*)- (3*R*,4*S*)-4-hydroxy melleins (**7** and **8**); and (3*R*)-6-hydroxymellein (**9**) were obtained as previously reported from *S. urbana* [45] and *P. chenopodiicola* [46], respectively. (3*R*)-6-methoxymellein (**10**) was prepared starting form compound **9** by reaction with an ether solution of diazomethane and MeOH.

### 4.2. Fungal Strains

*B. rosacearum* strain IRAN 4194C was obtained from vineyards of Paveh, Kermanshah Province, showing grapevines trunk diseases symptoms while the strain IRAN 4287C was obtained from oak trees showing charcoal canker and decline in Kermanshah Province (Gilan-e Gharb, Iran), respectively. DNA extraction, PCR, and maximum parsimony analysis were performed as previously reported [23]. ITS region of ribosomal DNA and a part of β-tubulin gene (*tub2*) were amplified for identification of the isolates. Sequences of both strains IRAN 4194C (ITS: MW786620) and IRAN 4287C (ITS: MZ359663; *tub2*: MZ362432) were deposited in GenBank. Their pathogenicity Koch’s postulates were followed under greenhouse conditions (22–28 °C). Fungal strains were deposited in collection of the Iranian Research Institute of Plant Protection (Tehrean, Iran) (IRAN).

### 4.3. Production, Extraction, and Purification of Secondary Metabolites

Both *B. rosacearum* strains (IRAN 4194C, IRAN 4287C) were inoculated and grown in stationary culture of potato dextrose broth (PDB) to produce secondary metabolites. The lyophilized culture filtrates (5 L) of *B. rosacearum* IRAN 4194C from grapevine re-dissolved in MilliQ H_2_O (1/10 of the initial volume, pH 5.5) and were extracted with EtOAc (3 × 500 mL). The combined organic extracts were dried (Na_2_SO_4_) and evaporated under vacuum, affording a residue of 746.0 mg, which was purified by SiO*_2_* column, and eluted with CHCl_3_/*i*-PrOH (9/1, *v*/*v*). Six homogeneous fraction groups were collected and the residue of the third fraction (431.0 mg) resulted to be a homogeneous oil, which was identified as *meso*-2,3-butanediol (**1**). The lyophilized culture filtrates (5 L) of *B. rosacearum* IRAN 4287C from oak trees were re-dissolved in MilliQ H_2_O in 1/10 of the initial volume, pH 7 and extracted with EtOAc (3 × 500 mL). The combined organic extracts were dried (Na_2_SO_4_) and evaporated under vacuum yielding a brown residue of 122.7 mg. This residue was purified by SiO_2_ column and eluted with CH_2_Cl_3_/*i*-PrOH (9/1, *v*/*v*). Seven groups of homogeneous fractions (F1–F7) were collected. The residue of F1 (14.5 mg) was further purified by TLC, using petroleum ether/acetone (7:3, *v*/*v*) as a solvent system, and yielding two bands. One of them resulted to be an homogeneous solid identified as nectriapyrone (**2**, 2.7 mg). The second band was further purified by TLC, using CH_2_Cl_3_/*i*-PrOH (9/1, *v*/*v*) as an eluent, affording two homogeneous solids identified as (3*R*)-5-methylmellein (**3**, 1.7 mg) and (3*R*)-5-methyl-6-methoxymellein (**4**, 1.5 mg). The residue of F3 (30.7 mg) was further purified by TLC, using petroleum ether/acetone (6:4, *v*/*v*) as a solvent yielding tyrosol as an amorphous solid (**5**, 3.5 mg).

#### 4.3.1. Meso-2,3-Butanediol (**1**)

Homogeneous oil, [α]^25^_D_ 0 (c 0.2); ^1^H NMR (CDCl_3_) δ: 3.80 (2H, q, *J* = 6.5 Hz, H-2 and H-3), 1.14 (6H, d, *J* = 6.5 Hz, Me-1 and Me-4); ^13^C NMR (CDCl_3_) δ: 70.8 (d, C-2 and C-3), 16.8 (q, C-1 and C-4). These data were in agreement with those reported by Gallwey et al. (1990) [26]. ESI MS (+) m/z: 73 [M + H−H_2_O]^+^.

#### 4.3.2. Nectriapyrone (**2**)

Amorphous solid, ^1^H NMR (CDCl_3_) δ: 6.69 (1H, q, *J* = 7.2 Hz, H-8), 6.09 (1H, s, H-5), 3.90 (3H, s, OMe), 1.94 (3H, s, Me-10), 1.89 (3H, s, Me-3), 1.84 (3H, d, *J* = 7.2 Hz, Me-9; ^13^C NMR (CDCl_3_) δ: 166.1 (s, C-4), 165.2 (s, C-2), 160.3 (s, C-6), 129.9 (d, C-8), 127.1 (s, C-7), 102.1 (s, C-3), 91.6 (d, C-5), 56.2 (q, OMe), 14.4 (q, C-9/C-11), 12.3 (q, C-11/C-9), and 8.8 (q, C-10). ESI-MS (+), *m*/*z*: 411 [2M + Na]^+^, 217 [M + Na]^+^, and 195 [M + H]^+^. These data were in agreement with those previously reported [9,33,34].

#### 4.3.3. (3*R*)-5-Methylmellein (**3**)

Amorphous solid [α]^25^_D_ -119 (c 0.2, CHCl_3_) (lit. Okuno et al., 1986 [33] [α]^20^_D_ -105 (c 0.36, CHCl_3_); ^1^H NMR (CDCl_3_) δ: 10.98 (1H, s, OH-8), 7.28 (1H, d, *J* = 8.4 Hz, H-6), 6.82 (1H, d, *J* = 8.4 Hz, H-7), 4.69 (1H, m, H-3), 2.95 (1H, dd, *J* = 16.5 and 2.0 Hz, H-4A), 2.72 (1H, dd, *J* = 16.5 and 11.9 Hz, H-4B), 2.19 (3H, s, Me-C5), 1.56 (3H, d, *J* = 6.5 Hz, Me-C3); ^13^C NMR (CDCl_3_) δ: 170.1 (s, C-1), 160.0 (s, C-8), 137.4 (d, C-6), 137.3 (s, C-4a), 134.5 (s, C-5), 115.2 (d, C-7), 108.3 (s, C-8a), 74.1 (d, C-3), 31.5 (t, C-4), 20.6 (q, Me-C-3), and 16.2 (q, Me-C-5). ESI-MS (+), *m*/*z*: 193 [M + H]^+^. These data were in agreement with those previously reported [35].

#### 4.3.4. (3*R*)-5-Methyl-6-Methoxymellein (**4**)

Amorphous solid [α]^25^_D_ -108 (c 0.2, CHCl_3_) (lit. de Alvarenga et al., 1978 [36], [α]^20^_D_ -98 (CHCl_3_); ^1^H NMR (CDCl_3_) δ: 11.36 (1H, s, OH-8), 6.38 (1H, s, H-7), 4.61 (1H, m, H-3), 3.85 (3H, s, OMe), 2.97 (1H, dd, *J* = 16.7 and 2.0 Hz, H-4A), 2.68 (1H, dd, *J* = 16.7 and 11.9 Hz, H-4B), 2.03 (3H, s, Me-C5), 1.56 (3H, d, *J* = 6.3 Hz, Me-C3); ^13^C NMR (CDCl_3_) δ: 170.2 (s, C-1), 164.6 (s, C-6), 162.9 (s, C-8), 137.6 (s, C-5), 137.1 (s, C-4a), 114.7 (s, C-8a), 115.1 (d, C-7), 74.8 (d, C-3), 55.7 (q, OMe), 31.9 (t, C-4), 20.9 (q, Me-C-3), and 10.6 (q, Me-C-5). These data were in agreement with those previously reported [34]; ESI-MS (+), *m*/*z*: 467 [2M + Na]^+^, 223 [M + H]^+^.

#### 4.3.5. Tyrosol (**5**)

White crystals, ^1^H NMR (CD_3_OD) δ 7.20 (d, *J* = 8.0 Hz, H-2 and H-6), 6.80 (d, *J* = 8.0 Hz, H-3 and H-5), 4.90 (s, OH), 3.80 (t, *J* = 6.4 Hz, H_2_-2'), 2.80 (t, *J* = 6.4 Hz, H_2_-1'); ^13^C NMR (CD_3_OD) δ: 156.5 (s, C-4), 133.6 (s, C-1), 133.0 (d, C-2,6), 118.4 (d, C-3,5), 65.4 (t, C-2'), 39.6 (t, C-1'). ESI-MS (+), *m*/*z*: 299 [2M + Na]^+^, and 139 [M + Na]^+^. These data are in agreement with those previously reported [9].

### 4.4. Leaf Puncture Assay

Compounds **1**–**10** were tested on *Quercus ilex* L. and *Hedera helix* L., using the leaf puncture assay at concentrations of 5 × 10^−3^ and 10^−3^ M. The compounds were dissolved in MeOH and then the solution was diluted with MilliQ H*_2_*O to reach the required concentration with a final concentration of MeOH at 4%. On the adaxial surface of the plant leaves, which were previously punctured with a sterile needle, a droplet (20 μL) of compound solutions was applied. The leaves were placed on the surface of a water-saturated filter paper in Petri dishes. The negative control was a solution of 4% MeOH in MilliQ H_2_O . The dishes were sealed with parafilm and incubated at 24 °C for 7 days in a temperature-regulated chamber. For each metabolite and plant species tested, three replications were performed. Seven days after treatment, necrotic lesion development was evaluated using a visual 0–3 scale (0 = no necrosis; 1 = slight necrosis; 2 = intermediate necrosis; 3 = severe necrosis).

### 4.5. Leaf Absorption Assay

Grapevine leaves (*Vitis vinifera* L.) were used for this assay. Briefly, the cuttings were placed in a tube containing the solutions of compounds **1**–**10** in 4% of MeOH/MilliQ H_2_O and tested at two concentrations (5 × 10^−3^ M and 10^−3^ M). Twelve hours after, the leaves were moved to another tube containing only MilliQ-H_2_O. The symptoms were evaluated after 48 h using a 0–3 scale (0 = no symptoms; 1 = slight symptoms; 2 = intermediate symptoms; 3 = complete wilting). A solution of 4% MeOH in MilliQ H_2_O was used as negative control. The experiment was carried out in triplicate.

## Figures and Tables

**Table 1:** Phytotoxic activity of compounds **1**–**10**.

		Bioassay		
		Leaf Absorption *^a^*	Leaf Puncture *^b^*	
Compound	Molar Concentration	*Vitis vinifera* L.	*Quercus ilex* L.	*Hedera helix* L.
**1**	5 × 10^−3^	3	2	0
	10^−3^	3	0	0
**2**	5 × 10^−3^	2	3	0
	10^−3^	2	1	0
**3**	5 × 10^−3^	2	1	0
	10^−3^	2	0	0
**4**	5 × 10^−3^	3	1	0
	10^−3^	3	0	0
**5**	5 × 10^−3^	2	3	0
	10^−3^	2	1	0
**6**	5 × 10^−3^	2	3	0
	10^−3^	1	1	0
**7**	5 × 10^−3^	1	1	0
	10^−3^	0	0	0
**8**	5 × 10^−3^	1	1	0
	10^−3^	0	0	0
**9**	5 × 10^−3^	3	1	0
	10^−3^	3	0	0
**10**	5 × 10^−3^	3	1	0
	10^−3^	3	0	0
**Control *^c^***	^−^	0	0	0

*^a, b^* Observations were made 2 and 7 days, respectively, after treatment. *^a^* Intensity of wilting symptoms are reported as: 3, complete wilting; 2, intermediate symptoms; 1, slight symptoms; 0, no symptoms. *^b^* Intensity of necrosis on leaves in leaf puncture assay are reported as: 3, severe necrosis; 2, intermediate necrosis; 1, slight necrosis; 0, no necrosis. *^c^* 4% MeOH in MilliQ H_2_O.

## Data Availability

Not applicable.
